# Drivers and deterrents of facility delivery in sub-Saharan Africa: a systematic review

**DOI:** 10.1186/1742-4755-10-40

**Published:** 2013-08-20

**Authors:** Cheryl A Moyer, Aesha Mustafa

**Affiliations:** 1 Global REACH, University of Michigan Medical School, 5115 Med Sci 1, 1301 Catherine St., Ann Arbor, MI 48109-5611, USA; 2 Department of Medical Education, University of Michigan Medical School, 5115 Med Sci 1, 1301 Catherine St., Ann Arbor, MI 48109-5611, USA

**Keywords:** Africa, Facility delivery, Maternal health service utilization, Systematic review

## Abstract

While the most important factors associated with facility-based delivery (FBD) have been explored within individual countries in Africa, no systematic review has explored the factors associated with FBD across sub-Saharan Africa. A systematic search of the peer-reviewed literature was conducted to identify articles published in English from 1/1995-12/2011 that reported on original research conducted entirely or in part in sub-Saharan Africa and included a primary outcome variable of FBD, delivery location, or skilled birth attendance (SBA). Out of 1,168 citations identified, 65 met inclusion criteria. 62 of 65 were cross-sectional, and 58 of 65 relied upon household survey data. Fewer than two-thirds (43) included multivariate analyses. The factors associated with facility delivery were categorized as maternal, social, antenatal-related, facility-related, and macro-level factors. Maternal factors were the most commonly studied. This may be a result of the overwhelming reliance on household survey data – where maternal sociodemographic factors are likely to be well-represented and non-maternal factors may be less consistently and accurately represented. Multivariate analysis suggests that maternal education, parity / birth order, rural / urban residence, household wealth / socioeconomic status, distance to the nearest facility, and number of antenatal care visits were the factors most consistently associated with FBD. In conclusion, FBD is a complex issue that is influenced by characteristics of the pregnant woman herself, her immediate social circle, the community in which she lives, the facility that is closest to her, and context of the country in which she lives. Research to date has been dominated by analysis of cross-sectional household survey data. More research is needed that explores regional variability, examines longitudinal trends, and studies the impact of interventions to boost rates of facility delivery in sub-Saharan Africa.

## Introduction

In 2011, nearly half of all the women who died due to pregnancy-related causes were from sub-Saharan Africa [1]. Skilled birth attendance (SBA) is one of the main interventions to combat such deaths, prompting the World Health Organization to advocate for universal SBA [2]. In many countries, encouraging women to deliver in facilities is the most practical way to boost rates of SBA.

In much of sub-Saharan Africa, fewer than half of women deliver their infants in health facilities [3]. The reasons are myriad, and understanding these factors is critical to identifying gaps in the existing research, planning interventions, and developing effective policies for addressing low facility-based delivery rates.

Three previous reviews of the literature have addressed facility-based delivery (FBD) [4-6], yet none were systematic, comprehensive, and focused on sub-Saharan Africa. The first review was not systematic, was conducted nearly 20 years ago, and the bulk of its references come from the mid-1980s [4]. This review addressed the factors that influenced the delay in deciding to seek care, the delay in getting to a health facility, and the delay in obtaining adequate care. The authors suggest that distance, cost, and quality of care are not sufficient to predict service utilization – other factors such as illness severity and socioeconomic status influence service use. This review resulted in what has come to be known as the Three Delays Model, perhaps one of the most commonly utilized conceptual frameworks in the maternal mortality literature. The second review focused on quantitative assessments of the impact of maternal health interventions on utilization [5]. Included in the review were a total of 30 quantitative studies from around the world, only 8 of which included data from sub-Saharan Africa. Say and Raine concluded that there is enormous variability in maternal health service utilization, and that utilization appears to be extremely dependent upon contextual factors [5]. The third review centered its assessment on references identified in the previous two reviews [6]. The authors used the literature to categorize determinants of facility-based delivery into four main themes: sociocultural factors, perceived benefit or need of skilled attendance, economic accessibility, and physical accessibility [6]. The authors conclude from their review that most research downplays perceived need and physical accessibility as significant barriers. Note that this review was not limited to any geographic region or any specific year range.

Given inherent differences between sub-Saharan Africa and much of the rest of the developing world, a review that explicitly focuses on sub-Saharan Africa is critical. In addition, a reconsideration of the domains of influence is also overdue. Thaddeus and Maine [4] see delays in care seeking as the crux of the issue around facility delivery. Say and Raine [5] do not posit a framework for understanding delivery location. Gabrysch and Campbell [6] see accessibility factors (including perceived need) and sociocultural factors as the most important drivers of decision making. This review attempted to explore the research literature in Africa to revisit the potential domains of influence over delivery location in sub-Saharan Africa.

We conducted a systematic review of the research literature of empirical studies addressing factors associated with FBD to: 1) document the research designs and data collection methodology used to explore factors associated with facility-based delivery in the published literature; and 2) identify the factors that are most commonly associated with FBD or SBA in sub-Saharan Africa.

## Materials and methods

### Search strategy

A systematic search of the peer-reviewed, published literature from 1995 – 2011 was conducted to identify the factors associated with delivery care in sub-Saharan Africa. Searches used: Ovid MEDLINE, EBM Reviews, International Pharmaceutical Abstracts, Journals@Ovid Full Text, CINAHL Plus with Full Text (EBSCO), PubMed, Africa-Wide Info, Psych Info, Global Health, Social Science Full Text, Google Scholar, BioMed Central, and African Journals Online. Initial searches were conducted on August 14 and September 5, 2011, and repeated on January 5, 2012.

The following key search terms were used in various combinations: maternal health services / utilization, developing country/ies, Africa, determinants or predictors, delivery services, facility-based delivery, facility delivery, institutional delivery, skilled birth attendance, skilled attendance, pregnancy. (Search strategy available upon request). Additional hand searching was conducted by reviewing the references of all retrieved studies.

### Study selection and data extraction

Studies were included in the review if they were published in a peer reviewed journal in English between January 1995 and December 2011, were conducted entirely or in part in sub-Saharan Africa, reported on the results of original research, and included a primary outcome variable of FBD, delivery location, or SBA. Articles needed to address determinants, predictors, or factors associated with delivery location. Review articles were not included. Due to an explicit emphasis on identifying empirically-tested associations, qualitative studies were excluded.

Study inclusion was determined in a multi-step procedure. First, bibliographic data and abstracts were evaluated for concordance with formal inclusion rules. Note that this first stage included the search term “developing country” or “developing countries”, but did not explicitly focus on African nations. At this first, most conservative decision point, studies were removed from further review if they were conducted in a western setting, but those conducted in developing countries were retained for closer inspection. Studies that clearly did not meet the remaining inclusion criteria were discarded.

The remaining studies were selected for full-text retrieval. Publications that did not present empirical data or otherwise did not meet inclusion criteria were discarded, but not before hand-searching the references. Full-text of studies identified from the references were retrieved as well. In a final step, the remaining studies were examined in detail to identify the final sample of studies meeting all inclusion criteria.

### Analysis and synthesis strategy

Given the variety of types of studies included in this systematic review – including descriptive and evaluative studies that ranged from simple bivariate analyses to complex multivariate modeling – a meta-analysis was neither possible nor appropriate. A table was created that listed all identified correlates of FBD. These correlates were grouped into categories: maternal factors, social factors, antenatal care-related factors, facility-related factors, and macro-level factors. The table also included a synthesis of findings indicating the direction of the relationship, the countries in which the research was conducted, and the citations associated with the research.

Each research study was coded independently by each author based a modified version of the STROBE statement [7]. The STROBE statement was used to develop a scale ranging from 0–34 points that covered such areas as study background, objectives, design, setting, participants, data collection methods, variables assessed, analysis methods, reporting of results, discussion of relevance to other literature, discussion of limitations, and inclusion of implications. Each item was scored on a 0–2 scale (0 = not included/addressed, 1 = somewhat included/addressed, 2 = clearly included/addressed). Total scores for each paper were compared across authors and averaged. Discrepancies of more than 4 points were discussed and consensus reached. Final averaged scores were divided into tertiles to determine the strength of the evidence, with those scoring in the lowest tertile providing ‘weak’ evidence, those scoring in the middle tertile providing ‘moderate’ evidence, and those in the top tertile providing ‘strong’ evidence.

## Results

**Figure 1:**
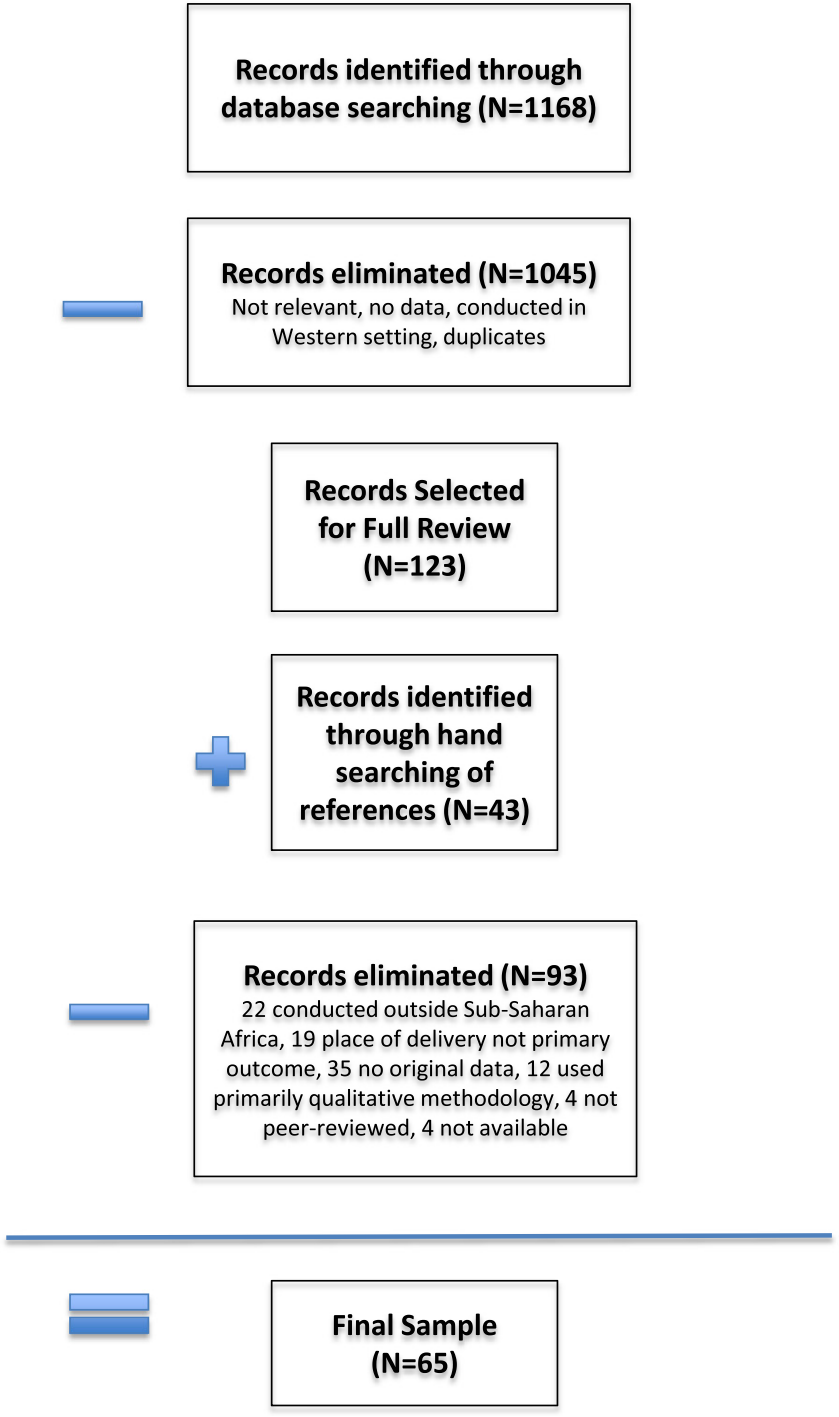
Flow diagram illustrating article selection and elimination.

1,168 citations were identified, of which 123 were retrieved for full-text review. Most of the 1,045 eliminated were excluded due to western setting, lack of original data, or a primary outcome measure other than place of delivery. Of the 123 articles retrieved for full-text review, an additional 43 studies were identified by searching the references, most of which were published in non-indexed, regional journals. Thus a total of 166 articles were identified for full text review. Upon reviewing the full text, another 93 were removed for such reasons as being conducted outside sub-Saharan Africa, place of delivery not being the primary outcome, not including original data, using primarily qualitative methods, not being peer-reviewed, or full text not being available. This left 65 published studies that met all inclusion criteria and for which data were extracted. (See Figure 1; Additional file 1).

Of the 65 articles, the mean STROBE score was 25.7 out of a potential 34 (range 14.5 – 32.0). The distribution was skewed toward higher scores, with 50% of all articles rating higher than 26 out of 34. In 57 out of 65 articles (87.6%), the coders agreed within 2 points on the article’s quality score. Only 4 articles showed discrepancy of 4 points (the maximum discrepancy found), and none of the discrepancies transcended the tertile cut points. In other words, scores between reviewers may have been 4 points apart, but either reviewer’s score would have put it in the same quality tertile. Three of the four articles in question were in the middle tertile, and one was at the highest tertile. (Additional file 1 illustrates the summary of all articles reviewed, including their quality ratings in tertiles).

**Pursuant to Aim 1** (*document the research designs and data collection methodology used to explore factors associated with FBD in the published literature*), all but 3 of the 65 published manuscripts included in this review were cross-sectional in nature. Ekirapa-Kiracho et al., 2011 [8], Penfold et al., 2007 [9], and Stanton et al., 2007 [10] were the only studies to include a longitudinal component, although none followed the same women over time. 58 out of the 65 studies (89.2%) reviewed relied upon population-based household surveys, including 20 that used national Demographic Health Survey data collected once every five years, and 6 that relied upon regional Health and Demographic Surveillance Site data, which are collected at least twice per year from small research outposts responsible for tracking the health and demographics of a surrounding population catchment area [11,12]. Nine out of 65 studies (13.8%) used medical records or facility assessment data, 4 used facility-based surveys of women, and 2 used Geographic Information System data (See Table 1).

**Table 1:** Data sources in empirical studies examining the factors associated with facility-based delivery in sub-Saharan Africa

**Type of data source**	Number of studies utilizing source*	**Percent of all studies**
Population-based / Household survey	58	84.0
- Demographic Health Survey Data	20	29.0
- Health and Demographic Surveillance Site Data	6	9.0
Medical records / Facility assessments	9	13.0
Facility-based Survey of Women	4	6.0
Published Literature	4	6.0
Geographic Information System Information	2	3.0

*Numbers total more than 65 because several studies used multiple data sources.

The sophistication of the data analysis varied widely. While 43 of the 65 studies (66%) included multivariate analysis, 20 (30.7%) included only descriptive statistics or a combination of descriptive statistics with bivariate associates explored. The remaining 2 studies utilized data compilation techniques to calculate rate ratios, odds ratios, or examine trends based on existing datasets [5,10,13].

**Pursuant to Aim 2** (*identify the factors that are most commonly associated with FBD or SBA in sub-Saharan Africa*), Tables 2, 3, 4 illustrate the factors identified in the literature as being associated with delivery location. These factors were divided into the following categories: maternal factors, social factors, antenatal care-related factors, facility-related factors, and macro-level factors.

**Table 2:** Maternal factors identified in relation to facility-based delivery rates in sub-Saharan Africa

**Maternal factor**	**Country in which it was studied**	**Direction of influence**	**Cites**
Maternal age	Botswana; Burkina-Faso; Ghana; Ivory Coast; Kenya; Malawi; Nigeria; Tanzania; 21 countries in Africa	Younger women more likely to deliver in a facility, except if very young (<18 years of age); inconsistently found significant	[14-22]
Maternal education	Botswana; Burkina Faso; Eritrea; Ethiopia; Ghana; Ivory Coast; Kenya; Malawi; Namibia; Nigeria; Tanzania; Uganda; multiple low-income, developing or African nations	Greater education is linked to higher levels of facility based delivery and skilled birth attendance	[14,16-21,23-47]
Religion	Ethiopia; Ghana; Nigeria; Uganda	Those who practice traditional or Muslim religions in some countries are less likely to deliver in a facility, although finding is not universal	[14,16,35,39,40,42,48]
Ethnicity	Burkina Faso; Ghana; Kenya; Nigeria; Tanzania; Uganda	Ethnicity has an inconsistent relationship with FBD. In some settings ethnic minorities are more likely to seek FBD, in other settings ethnic minorities are less likely to seek FBD	[25,27,28,31,33,40,42,49-51]
Region / province of residence	Ghana; Kenya; Rwanda; Tanzania; Uganda	Region, province of residence has an inconsistent relationship with FBD. In some nations there are strong regional and provincial differences, even after controlling for rural/urban status. In other nations, regional differences are largely explained by rural/urban or socioeconomic status	[16,25,34,35,40,50,52,53]
Urban / Rural residence	45 developing countries; Botswana; Eritrea; Ethiopia; Ghana; Kenya; Mali; Namibia; Nigeria; Rwanda; Senegal; South Africa; Tanzania	Urban women more likely to deliver in a facility than rural women; however poverty is tightly linked to urban / rural status	[13,14,19,25,26,31,33-35,39],[43,46-48,52,54-56]
Wealth / SES / economic variables	31 countries in Africa; 45 developing countries; Botswana; Burkina Faso; Ghana; Kenya; Namibia; Nigeria; Rwanda; Tanzania; Uganda	Poorest women least likely to use delivery services; FBD seen as causing financial hardship; inequalities across wealth groups smallest in countries with highest female literacy rates	[13,19,26,28-33,35-37,40,42,43],[47,50,52,57]
Maternal employment (status / occupation)	Eritrea; Ethiopia; Ghana; Kenya; Nigeria; Zimbabwe	Maternal employment positively linked to FBD	[16,28,42,46,58]
Health insurance coverage	Ghana; Kenya; Mali; Nigeria; Rwanda; Senegal; Tanzania	Insurance coverage, fee exemptions linked to greater FBD rates; Membership in a voluntary community-based health insurance program was linked to increased FBD	[9,17,52,56,59,60]
Parity / birth order	73 countries; Botswana; Burkina Faso; Ethiopia; Ghana; Ivory Coast; Kenya; Malawi; Nigeria; Tanzania	Higher parity, lower likelihood of FBD; No previous births linked to FBD; Birth order higher than 4, FBD less likely; Lower in the birth order, FBD more likely	[10,14,15,17,19,20,22,25],[27-29,31,39,42,45,50,60,61]
Marital status	Ethiopia; Kenya; Tanzania; Uganda; Zimbabwe	Marital status linked to FBD in some studies, not linked in others	[21,24,31,39,58]
Polygamous union	Ghana; Senegal	Less likely to have FBD	[35,62]
Empowerment / Autonomy	31 countries in Africa; Eritrea; Ethiopia	Women with highest levels of empowerment most likely to seek FBD, have SBA; Other research suggests autonomy and wealth interact but autonomy alone is insufficient	[28,32,46]
Attitude toward importance of FBD / perceived need	48 developing countries; Nigeria; Tanzania	"Childbirth is natural" - no need for FBD; "FBD is important" linked to higher utilization	[15,57,60,61,63]
Attitude toward skills of doctor vs. TBA	Kenya; Tanzania	Perceived similarity of skilled vs unskilled attendants linked to lower FBD rates	[45,61]
Embarrassment / fear of being shamed	Tanzania	Not having clean clothes for self or baby, embarrassment of poverty linked to lower FBD	[50]
Discussion with male partner on place of delivery	Tanzania	Discussion with male partner linked to higher FBD rates	[21]
Knowledge of pregnancy risk factors / safe delivery	Kenya; Tanzania	Greater knowledge linked to higher FBD rates	[21,45]
Completion of a birth plan	Uganda	Completion of a birth plan linked to FBD	[24]
Concept of abnormal vs. normal pregnancy	Nigeria	"Normal" pregnancies mean home delivery is preferred	[63]
Having means of transport to facility / vouchers for transport	Ghana; Mali; Senegal; Uganda	No transport means FBD less likely	[8,62,64,65]
Quality of previous delivery	Senegal	Poor quality previous delivery means less likelihood of FBD on subsequent deliveries	[62]
Location of previous delivery	Kenya; Uganda	Location of previous delivery predicts subsequent delivery location	[40,45]
Pregnancy wantedness	Kenya	Desired pregnancies more likely to be delivered in facility	[25,28,30]
Birth complications / perceived problems	Tanzania; Zimbabwe	When problems arose, women reported desire to be in a facility; Complications during previous pregnancy predictive of FBD	[50,58]
Use of herbal drugs in pregnancy	Nigeria	Use of herbal drugs associated with lower FBD rates	[42]
Desire to appear modern	Tanzania	Greater desire to appear modern linked to greater FBD	[50]
Fear of episiotomy	Swaziland	Fear of episiotomy linked to lower FBD	[66]
Precipitate Labor	Ghana; Swaziland	Decreased likelihood of FBD	[34,66]
Use of maternity waiting homes	Zimbabwe	Increased likelihood of FBD	[58]

**Table 3:** Social factors identified in relation to facility-based delivery rates in sub-Saharan Africa

**Social factor**	**Country in which it was studied**	**Direction of Influence**	**Cites**
Non-male household head	Kenya	Increased likelihood of FBD	[59]
Husband's occupation	Kenya; Nigeria	Non-farmers have higher rates of FBD	[18,59]
Husband / partner's education	Eritrea; Ethiopia; Kenya; Nigeria	Greater husband's education, greater FBD	[17,27,46]
Small family norm (community level)	Nigeria	Small family norm linked to greater use of SBA	[33]
Stigma / risk of gossip / onlookers	Uganda	FBD puts women at risk of gossip, stigma, social devaluation	[67]
Living in a socioeconomically disadvantaged neighborhood	Nigeria	Linked to lower likelihood of FBD	[17]
Permission from husband, TBA, mother, or mother-in-law	Gambia	Needing permission linked to lower likelihood of FBD	[68]
Social influence of others	Tanzania	Attitudes of others encourage / discourage FBD rates	[61]
Village level: % of village who agree that FBD is important	Tanzania	Higher percent linked to greater FBD rates	[60]
Village level: % of village who rated local facility as "excellent"	Tanzania	Higher percent linked to greater FBD rates; Unrelated in Mills study	[60,65]
Village level: % of village who attended 4+ ANC visits	Tanzania	Higher percent linked to greater FBD rates	[60]
Village level: % of village who agreed doctors and nurses have good skills	Tanzania	Higher percent agreeing linked to higher FBD	[60]
Village level: % of village who agreed TBAs have good skills	Tanzania	Higher percent agreeing TBAs have good skills linked to lower utilization of FBD	[60]
Community perception of access to nearest facility	Ghana	Higher perception of access linked to higher FBD rates	[65]
Traditional views on delivery and motherhood	Swaziland	More traditional views yield lower FBD rates	[66]

**Table 4:** Antenatal care (ANC), facility, and macro-level factors identified in relation to facility-based delivery in sub-Saharan Africa

**Antenatal care factor**	**Country in which it was studied**	**Direction of influence**	**Cites**
Attended ANC	Kenya	ANC attendance linked to higher likelihood of FBD	[31]
Timing of first ANC visit (early onset of ANC)	Tanzania; Ghana	Earlier ANC initiation linked to greater likelihood of FBD; Later ANC linked to FBD	[49,50]
Number of ANC visits	Burkina Faso; Ghana; Ivory Coast; Kenya; Malawi; Tanzania	Fewer ANC visits linked to lower likelihood of FBD; 3+, 4+ visits linked to higher rates of FBD	[14,15,25,27-30,51]
Saw doctor at ANC	Ghana	Seeing a doctor at ANC linked to greater FBD	[49,54]
Quality of ANC	Ghana	Higher perceived quality linked to greater FBD	[54]
Being advised to deliver in a facility during ANC	Ghana; Kenya; Tanzania	Higher likelihood of FBD	[21,28,30,34,50]
FACILITY FACTOR			
Distance to facility	Burkina Faso; Ghana; Kenya; Malawi; Mali; Nigeria; Senegal; Tanzania; Uganda; Zambia	Greater distance, lower likelihood of FBD	[15,21,22,25,34,36,44,48],[50,51,59,69,70]
Cost	Ghana; Nigeria; Uganda	Greater cost associated with lower likelihood of FBD	[48,63-65,70,71]
Promptness of care	Nigeria	Perception of promptness of care linked to greater utilization	[48]
Perceived quality of delivery care	Ghana; Nigeria; Tanzania	Individual perceptions about higher quality of care linked to higher FBD rates. One study showed no relationship between community perceptions of quality and individual FBD	[34,48,60,65,71]
Presence of any provider, presence of OB/GYN, 24-hour availability of provider	Nigeria	Higher likelihood of FBD	[48,71]
Availability of medicine, equipment, emergency obstetric care	Nigeria; Tanzania; Uganda; Zambia	Increased FBD when medicine, equipment, higher level of emergency obstetric care available	[48,53,69,72]
Staff attitudes / behavior	Nigeria; Swaziland; Tanzania; Uganda	Negative staff attitudes, abusive treatment at hands of HCPs related to lower FBD	[48,63,66,67,72]
Culturally unacceptable	Nigeria; Swaziland	Less likely to deliver in a facility	[63,66]
Previous delivery with male provider	Senegal	Less likely to deliver in a facility	[62]
Electricity, running water, radio communication at facility	Uganda	Presence of infrastructure linked to higher FBD rates	[53]
MACRO-LEVEL FACTOR			
Government share of health care spending	42 low-income countries	Greater percentage of government spending, greater likelihood of SBA	[23]
Female literacy rates (education)	42 low-income countries	Higher rates of female literacy in a country associated with higher rates of SBA	[23,26]
Total health expenditures per capita	42 low-income countries	Higher total health expenditures per capita associated with higher rates of SBA	[23,37]
Gross national income per capita	21 sub-Saharan African countries	Higher gross national income per capita linked to FBD	[20]

Table 2 addresses more than 30 different maternal factors that have been explored in sub-Saharan Africa pursuant to FBD, the most common being maternal education, urban/rural status, and socioeconomic status. A host of additional maternal factors were found to be associated with FBD, including parity, perceived need for FBD, having means of transport to a facility, previous delivery location, and perceived complications. Many of these variables have a consistent and predictable relationship with facility-based delivery – such as greater education and higher socioeconomic status generally predicting greater utilization of FBD services. Others appear to have differential effects, based upon the study locale, design, or population. For example, marital status appears to be linked to facility delivery in some studies, yet not in others. Female autonomy appears to be associated with greater facility delivery rates in some studies, yet other studies indicate a strong interaction effect with wealth, suggesting that women’s autonomy in the absence of material resources is insufficient to boost facility utilization.

Table 3 illustrates 15 different social factors found to be associated with FBD. Social factors include such things as non-male household head, husband’s occupation, husband’s education, small family norm, living in a socially disadvantaged neighborhood, or needing permission to go to a facility. Relative to the maternal factors described in Table 2, social factors appear to be much less studied, with 12 unique studies accounting for data pursuant to 15 identified social factors. The social factors most commonly cited as related to FBD include husband’s education and occupation, as well as a village-level variable regarding the percent of the community rating the local facility as excellent. In terms of direction of influence, women with more educated husbands or husbands in non-agricultural occupations are more likely to deliver in a facility. In addition, women in communities that rank their local facility as ‘excellent’ are more likely to deliver in a facility.

Table 4 illustrates the role antenatal care (ANC) may play in influencing facility based delivery. With one exception, the results suggest that all elements of ANC are linked to greater utilization of FBD services. Akazili et al. [49] found that in northern Ghana, women who presented for ANC during the third trimester were more likely to deliver in a facility than women presenting earlier. The authors speculate that may be a result of women with complications presenting late for ANC and being strongly encouraged to deliver at a facility.

Table 4 also illustrates the numerous facility-related factors that may influence whether women choose to deliver at home or in a facility. In this category, distance to facility is the most common factor studied and cited as a deterrent to FBD. In looking at the number of studies citing each factor, cost, perceived quality of care, and staff attitudes and behavior are the next most common facility-related factors identified in the literature.

Finally, Table 4 illustrates some of the macro-level factors that appear to be associated with FBD and SBA rates. SBA appears to be higher in countries in which the government spends a larger percentage of its spending on health and in which there is higher total health expenditure per capita. In addition, countries with higher rates of female literacy are likely to have higher rates of SBA than countries with lower female literacy rates.

Out of the 43 manuscripts reviewed that used multivariate modeling, 37 reported one or more models in their results in sufficient detail to allow for comparison across studies. “Full” models ranged from those that included only three variables (e.g. Kruk et al., 2007 [23]; Mulogo et al., 2006 [24]; Penfold et al., 2007 [9]) to those that included 15 or more variables (e.g. Gabrysch et al., 2011 [69]; Spangler and Bloom, 2010 [50]; Stephenson et al., 2006 [14]). Across the multivariate models and among those studies deemed to be of moderate or strong quality, the factors that showed the greatest consistency in their association with FBD were maternal education, parity, household wealth, urban residence, distance to the nearest facility, and number of ANC visits. Table 5 illustrates those studies in which multi-variate models explored some or all of those factors, indicating the consistency of the findings across studies and across models (See Table 5). Only one of the published studies in this review included a model with all of these variables, finding all to be statistically significant [25]. Beyond these key variables, the host of additional factors studied in multivariate analysis were not consistently found to be associated with FBD. These included age-related variables, ethnicity, religion, marital status, partner’s occupation, previous health-related factors, and women’s autonomy.

**Table 5:** Multivariate models including education, parity, urban status, and wealth as correlates of facility delivery

	**Aremu et al., 2011 - Nigeria**	**Babalola et al., 2009 - Nigeria**	**Hong et al., 2011 - Rwanda**	**Letamo et al., 2003 - Botswana**	**Magadi et al., 2000 - Kenya**	**Smith, Sulzbach, 2008 - Mali**	**Smith, Sulzbach, 2008 - Ghana**	**Stephenson et al., 2006-Malawi**	**Stephenson et al., 2006 - Kenya**	**Stephenson et al., 2006 - Tanz.**	**Stephenson et al., 2006 - B. Faso**	**Stephenson et al., 2006 - Ghana**	**Stephenson et al., 2006 - I. Coast**
Maternal age	*			*		*	ns	*	*	*	*	*	*
Age at last birth		ns	*										
Maternal education	*	*	*	*	*	ns	ns	*	*	*	*	*	*
Partner's education	*												
Age x parity interaction				*									
Parity / birth order	*	ns	*	*	*	ns	ns	*	*	ns	ns	*	*
Marital status				*				*	*	ns	ns	ns	*
Maternal occupation	*		*										
Religion						ns	ns	ns	*	*	ns	*	ns
Ethnicity		*			*	ns	ns						
Region	ns	ns	ns		*								
Rural / urban	ns	*	*	*	*	ns	*	*	ns	*		*	
Insurance	*		*			*	ns						
Household wealth / SES	*	*	*	*	*	ns	*	*	*	*	*	*	*
Neighborhood SES / slum residence	*												
Pregnancy intendedness					*								
Attitude toward family planning		ns						*	*	*	ns	ns	ns
Exposure to family planning info								*	*	*	*	*	ns
History of newborn death						ns	ns						
Ideal family size		ns											
Prevalence of small family norm		*						*	ns	ns	ns	*	ns
Media saturation		*											
Ever used modern contraception					*								
Previous hospital delivery								*	*	*	*	*	*
Number of antenatal care visits					*			*	*	*	*	*	*
Location / distance to nearest facility					*								
Percent of women w/secondary + education								*	*	ns	*	*	ns
Rainfall category of Primary Sampling Unit (PSU)								ns	*	ns	ns	ns	ns
Percent of women in PSU w/1+ prior FBD								*	ns	*	*	*	*
Total # of variables in model	10	11	8	7	10	9	9	15	15	15	14	15	14

*p<0.05.

## Discussion

In summary, the vast majority of the empirical research conducted on FBD in sub-Saharan Africa is cross-sectional in nature and relies upon data from household surveys. In addition, the literature to date is variable in its quality and analytical sophistication. Maternal factors – especially sociodemographic factors – appear to have been the most frequently studied and are among the factors most commonly linked to FBD rates. This may be a result of the overwhelming reliance on household survey data – where maternal sociodemographic factors are likely to be well-represented and non-maternal factors may be less consistently and accurately represented. Nonetheless, a host of non-maternal factors spanning social, ANC, facility-related, and macro-level factors emerge from this literature and appear to be associated with FBD rates in sub-Saharan Africa.

One critical gap identified in this review of the literature is studies with a longitudinal design. In many studies, data are collected from women well after delivery, and women are queried about their decision-making regarding delivery location. Such a design asks women to reflect back on the reasons that compelled them to stay home or deliver in a facility. While this may be the most practical and feasible way to gather such information, it may be subject to recall bias and is likely to be influenced by women’s experiences during delivery. In contrast, much could be learned if attitudinal and behavioral data were collected from women throughout their pregnancies, further examining those data in the context of their ultimate delivery location.

Few studies to date have explored regional variability in FBD in a meaningful way. While many studies report regional differences, none adequately explored the factors underlying those differences beyond attributing them to socioeconomic status, rural/urban differences, or ethnicity. What is it about ethnicity, for example, that predisposes some women to deliver at home versus delivering in a facility? Is ethnicity a proxy for education, or socioeconomics, or rural/urban status? And while socioeconomic status is seen as inextricably linked to FBD rates, why does socioeconomic status appear to be less important in countries with higher female literacy rates? [26].

Another gap in the FBD literature is the dearth of intervention studies. It is possible that there are simply not enough intervention studies underway or completed in the region to be able to generate peer-reviewed publications. It is also possible that the interventions underway focus on primary outcomes aside from FBD and thus were not picked up in this review. For example, Bellows et al. [73] conducted a systematic literature review regarding the use of vouchers to encourage reproductive health service use that was not discovered through this review. Yet that review included only three studies conducted in Africa, one on sexually transmitted infection care and maternity services in Uganda [74] and two on family planning in Kenya that included a maternity services component [74-76]. It is also possible that research capacity in many of the developing nations of sub-Saharan Africa is such that translating research results into submitted publications is hampered by limited human resources. Regardless, intervention studies are needed to determine how to successfully boost FBD rates in sub-Saharan Africa.

Research literature to date has relied heavily on household surveys, especially the Demographic Health Surveys conducted every five years in many developing countries. While such data are plentiful and readily available for analysis, it is important to recognize their limitations. First, household surveys are typically conducted through verbal interviews with women and/or heads of household, a format which can increase the risk of social desirability bias. Household surveys also limit the number and type of questions that can be asked, which may affect the ultimate conclusions drawn. For example, in this review 11 studies relying upon household data found that ANC use, frequency, and perceived quality are associated with a greater likelihood of FBD [14,15,25,27-31,50,51,54]. This finding contradicts some of the qualitative literature suggesting that women who are told they have “normal” pregnancies during antenatal care assume they will have “normal” deliveries and thus do not need to deliver in a facility [77,78]. While these two seemingly discrepant findings may both be valid, note that the latter could not have been detected in a cross-sectional household survey.

In addition, household surveys are not ideal for measuring social norms, social networks, individual integration into social networks, availability of social support, community-level attitudes toward health behaviors, or decision-making patterns within extended families – all of which have the potential to vastly improve understanding of FBD in sub-Saharan Africa. Thus, another critical gap in the literature includes studies that move beyond household surveys to examine the social factors influencing delivery location.

Finally, this review illustrates the enormous variability with regard to the analysis of data associated with FBD. Nearly a third of the studies in this review were limited to descriptive and bivariate statistics. While such studies may provide insights into which variables require further research, multi-level and multi-variable modeling is important to advancing this literature. Nonetheless, caution is warranted: Results from sophisticated analytical procedures will only reflect the data being included in the models; and as described, key social and community-level components of the equation may be missing altogether.

This systematic review of the literature builds upon the previous reviews in several important ways. First, it focuses entirely on sub-Saharan Africa, explicitly including African journals. This is a departure from previous reviews. Thaddeus and Maine’s 1994 review, while generally focused on maternal mortality in Africa, included articles from Central and South America and across Asia and the Middle East [4]. Similarly Say and Rayne’s 2007 review [5] included only 8 articles from Africa, and Gabrysch and Campbell’s 2009 review [6] – which was based upon Thaddeus and Maine’s and Say and Rayne’s reviews – included studies across Latin America, Asia, and the Middle East. While such inclusivity might have been helpful at a time when there was comparatively little written about barriers to facility delivery, it is not nearly as useful today in planning interventions that speak to the local context. The review presented here focuses exclusively on the issues pursuant to the sub-Saharan African context, something that has been sorely missing in the published literature. In addition, this review sought to include original research from the African sub-continent that was not published in mainstream western literature. This has complicated the search strategy for this review, and admittedly, it has increased the variability of the quality of studies reviewed. However, many of the articles retrieved from the African journals included in this search have shed valuable light on the phenomenon of FBD that might have otherwise gone unnoticed.

This review challenges assumptions made in previous reviews about how to categorize the factors associated with FBD. This review proposes that the factors associated with facility delivery fall into five different categories: maternal, social, antenatal, facility-related, and macro-level factors. This categorization suggests a much broader lens than those posited previously. Maternal factors have always been a focal point of policy and programming, but social factors have received much less attention. Yet social factors such as community attitudes toward facility delivery are likely an important intervention point. This review also suggests that women’s experiences during ANC (and with the facility itself) may be extremely important in influencing future maternity service use. As such, the facility and those who staff it may be an important target of future interventions. In addition, researchers and policy makers must be mindful of the regional and national context. Low FBD rates may be a downstream effect of lack of national emphasis on education of girls, for example.

Despite its strengths, this review has several limitations worthy of note. First, the review was limited to articles published between 1995 and 2011. It is possible that having broadened the years of publication, the results may have been slightly different. Second, the review was limited to articles published in English and available via English-language search engines. This is an important limitation, given the number of Francophone countries in Africa and the likelihood that research coming from those countries may tell a very different story than those coming from English-speaking nations. By design, this review also focused upon quantitative studies that could provide statistical assessments of associations. The results may have been different – albeit perhaps more difficult to compare – if qualitative studies were also included in the assessment. This review was conducted by a small team of researchers, which may have affected the interpretation. The author was assisted in creating and implementing the search strategy by a master’s trained global health librarian, and the quality of the articles was judged by the author and a master’s level research associate. This small team was efficient, but it is possible that a larger team may have interpreted the literature slightly differently.

## Conclusions

In conclusion, FBD is a complex issue that is influenced by a host of factors, including characteristics of the pregnant woman herself, her immediate social circle, the community in which she lives, the facility that is closest to her, and context of the country in which she lives. While multivariate analysis suggests that across sub-Saharan Africa, maternal education, parity, rural / urban residence, household wealth, distance to the nearest facility, and number of ANC visits are the factors most strongly and consistently associated with FBD, the literature suggests that dozens of additional factors appear to contribute to FBD rates in both bivariate and multivariate analyses. Further research is needed to determine the relative strength and the replicability of such findings, given the enormous variability seen within and across the nations of sub-Saharan Africa. In addition, longitudinal and intervention research are needed to advance understanding of how best to increase FBD in sub-Saharan Africa.

## Competing interests

Both author declares no competing interests.

## Authors’ contributions

CM conceived of the idea, planned the study, and conducted all searches. CM and AM worked together to determine whether studies met inclusion criteria and then both authors independently assessed each selected article for quality. CM drafted the initial manuscript and AM participated in critical review and revision. All authors read and approved the final manuscript.

## Supplementary Material

Additional file 1 **Characteristics of final sample of 65 studies included in systematic review **[79-82]**.** Click here for file
