# Supplementary material for: Drivers and deterrents of facility delivery in sub-Saharan Africa: a systematic review
Source: Reprod Health. 2013 Aug 20;10:40. doi: 10.1186/1742-4755-10-40 (PMC3751820; doi:10.1186/1742-4755-10-40)
Supplement: Additional file 1 — Characteristics of final sample of 65 studies included in systematic review [79-82]. [file 1742-4755-10-40-S1.doc]

**Additional file 1. Characteristics of final sample of 65 studies included in systematic review**

| **Author, Year** | **Country** | **Study Design** | **Data Source, Year** | **Sample size, description** | **Main Predictor Variables assessed** | **Main Outcome Variables Assessed** | | **Analysis Method** | **Main Findings per Delivery Location** | **Quality Tertile** |
| --- | --- | --- | --- | --- | --- | --- | --- | --- | --- | --- |
| Adanu, 2010 [54] | Ghana | Cross-sectional | Ghana Demographic Health Survey, 2003 | 2777 women aged 15-49 with at least 1 pregnancy between 1999-2003 | ANC provider, services done during ANC, rural/urban residence | Place of delivery, who attended delivery | | Descriptive statistics, bivariate comparisons | In 1999-2003, 49% of women delivered in their own home, 7.2% in someone else's home, 26.2% delivered in a gov't hospital, 7.8% at gov't health center; 6.5% attended by doctor, 40.5% attended by nurse/midwife, 30.3% attended by TBA. Predictors of delivery by doctor: urban residence, saw doctor at ANC. Higher quality ANC linked to SBA. Rural residence linked to TBA delivery. | weak |
| Addai, 2000 [16] | Ghana | Cross-sectional | Ghana Demographic Health Survey, 1993 | 4562 women aged 15-49 who gave birth within past 3 years and live in a rural area | Ethnicity, religion, respondent's education, age, age at marriage, living children, region of residence, occupation | 1) Use of doctor for ANC, 2) 4+ ANC visits, 3) Place of delivery (hospital vs. home), 4) Participation in family planning | | Logistic multiple regression | Women with at least secondary education are more likely to deliver in a facility (61.5%) than those with primary or junior schooling (38.9%) or no schooling (16.5%) ; Large regional variability across Ghana in FBD rates ; Traditional religion associated with lower rates of FBD ; In multivariate analysis, age, religion, education, occupation, and region were strongest predictors. | moderate |
| Ahmed et al., 2010 [32] | Developing Countries | Cross-sectional | Demographic Health Surveys from 31 countries, 1998-2006 | Women aged 15-49 from DHS data in 31 countries | Wealth quintile, education (complete primary vs. no or incomplete primary), composite score of women's autonomy | 1) Use of modern contraception, 2) 4+ ANC visits, 3) SBA | | Logistic regression + meta-analytic techniques | Women experiencing inequities in the 3Es (economic, educational, empowerment status) in the 31 countries (21 in Africa) are less likely to use health services in general. Poorest women are 94% less likely to use SBA. Women who completed primary education are 5 times more likely to have SBA. Women with the highest empowerment score are 1.31 times more likely to have SBA. (Women's empowerment is the least strong factor.) | moderate |
| Akazili et al., 2011 [49] | Ghana | Cross-sectional | Household panel survey from nothern Ghana, 2002 | 4375 women who reported on last delivery in 2002 survey administration | Source of antenatal care, # of ANC visits, timing of onset of ANC, age, education, marital status / type, belief in spirit children | Place of delivery (likelihood of home delivery) | | Multivariate logistic regression | 71% delivered at home, 25% delivered at a hospital/clinic, 4% delivered at a health center. In multivariate model, compared to having ANC with a doctor, no ANC provider = 9.7 x greater likelihood of delivering at home, ANC by nurse/midwife = 2.3 x higher odds of delivering at home, ANC by CHO = 5.2 x higher odds of home delivery. Greater # of ANC visits and later stage of ANC initiation linked to lower likelihood of home delivery. Ethnicity significantly related, with Nankanis less likely to deliver at home. Age, education not significant. | moderate |
| Aremu et al., 2011 [17] | Nigeria | Cross-sectional | Nigerian Demographic Health Survey, 2008 | 15,162 ever-married women | Individual level: age, education, occupation, place of residence, insurance, parity, partner's occupation, household wealth index; Neighborhood level: MD-provided ANC, geographic region, region of residence, neighborhood socioeconomic disadvantage | 1) Birth at gov't hospital; 2) Birth at private hospital; 3) Birth at home | | Multilevel discrete choice modeling | 71% of population gave birth at home. Gov't facility use over home use predicted by greater education, greater partner's education, health insurance coverage. Less than 34 years of age and birth order higher than 4 less likely to use gov't facility. "Living in a highly socioeconomically disadvantaged neighborhood is associated with greater use of home for childbirth than gov't facilities, even after controlling for women's socioeconomic position and that of her household." | strong |
| Babalola and Fatusi, 2009 [33] | Nigeria | Cross-sectional | National HIV/AIDS and Reproductive Health Survey, 2005 | 2148 women who had delivered during the 5 years preceding the survey | Individual level: education, age at last birth, ethnicity, child's birth order, attitudes toward family planning, ideal family size enumerated; Household level: SES; Community level: rural/urban, media saturation, small-family norm; State level: average # of people per PHC in state of residence | 1) ANC; 2) SBA; 3) Post-natal care | | Multi-level analytic methods + state-level random effects | 43.5% of sample had SBA at the most recent delivery. Individual level predictors of SBA: education, ethnicity, enumerated ideal family size; Household level: SES; Community level: urban status, media saturation, small family norm; State level: greater # people per PHC decreases likelihood of SBA. | moderate |
| Bazant et al., 2009 [27] | Kenya | Cross-sectional | World Bank and African Pop and Health Research Center of Nairobi Household Survey, 2006 | 1926 women from 2 informal settlements in Kenya | Women's slum residence, age, education, ethnicity, marital status, occupation, # of children, pregnancy intendedness, perception of complications, C-section, wealth of household, unaccompanied mobility | Location where women delivered: 1) home, home of TBA; 2) private health care facilities, 3) gov't health facilities | | Multivariate, multinomial logistic regression | Overall, 45% gave birth in a private facility, 21% at a gov't facility, 34% w/o trained professional; Predictors of place of birth: education (gov't hospital), more children (home delivery), ethnic group variability, more husband/partner education (more likely to deliver in a facility), fewer ANC visits (less likely to deliver at a gov't hospital). | strong |
| Cotter et al., 2006 [79] | Kenya | Cross-sectional | Medical records at Kikoneni Health Center, Kenyan National Census Data, Mar 2001- Mar 2003 | 994 women at Kikoneni Health Center who attended ANC, estimated # of births in region | Of women who had ANC at KHC, what percentage delivered there? Of expected number of deliveries in the region in the time period (based on census data), what percentage had recorded skilled birth attendance coverage? | | | Descriptive statistics and extrapolation | 7.4% of women who gave birth at KHC had ANC there (74/994); 74 SBA deliveries were recorded in a period in which 1373 births were estimated to have occurred (SBA coverage ~ 5.4%). | weak |
| Cronje et al., 1995 [55] | South Africa | Cross-sectional | Household survey, 1991 | 237 rural black women, 168 urban black women who had delivered or aborted within previous year | Age, ANC utilization | | Place of delivery, who supervised delivery | Descriptive statistics | 60% of rural women delivered at home and 37% delivered in a hospital (supervised by family member in 32% of cases, nurses in 31% of cases, traditional midwives in 26% of cases, doctor in 9% of cases); 23% of urban women delivered at home and 67% delivered in a hospital (supervised by nurses (66%), family members (14%), doctors (12%), and traditional midwives (5%) | strong |
| Danforth et al., 2009 [61] | Tanzania | Cross-sectional | 2-stage representative cluster sampled household survey, 2007 | 826 couples in Kasulu district of Tanzania, wife had delivered in past 5 years | Woman's age, man's age, wealth status, education of woman, # of children, location of nearest facility, perceptions of importance of FBD and skills of doctors vs. TBAs | | Place of delivery, facility (yes / no) | Logistic multiple regression | 61.3% of women (506) delivered at home. Multivariate: 2+ children decreases odds of FBD, disagreement on importance of FBD decreases odds of FBD; Associated with FBD: partners agree on importance of FBD, agree on skills of doctor being better than TBA. When partners disagreed, opinion of woman was more influential. | strong |
| De Allegri et al., 2011 [51] | Burkina Faso | Cross-sectional | 3-stage cluster sampled random household survey in rural northwest Burkina Faso, 2009 | 435 women who reported a pregnancy in the past 12 months | Woman's age, religion, ethnicity, literacy, marital status, history of miscarriage, parity, household head's literacy, household head's age | | ANC utilization, Delivery in a facility | Multivariate logistic regression | 7.2% of women delivered in a facility; Ethnicity, living within 5km of a health facility, having attended at least 3 ANC visits all linked to higher likelihood of FBD. | strong |
| Ejembi et al., 2004 [63] | Nigeria | Cross-sectional | Household survey of women of reproductive age (10-49), year not stated | 655 currently married women in rural north-western Nigeria | Woman's age, education, occupation, fertility history, attitudes toward contraception, ANC, and FBD | | 1) Contraceptive knowledge and use, 2) ANC utilization, 3) Facility-based delivery | Descriptive statistics, bivariate comparisons | 576 women had a history of pregnancy and at least 1 full-term delivery; 9.9% of last deliveries occurred in a hospital (down from 1986 numbers); 15% of births had SBA; 42% no attendant, 40% had untrained attendant, and 3% had a trained TBA. Of 524 who delivered at home, reasons given included: culturally unacceptable (37%), hospitals are too far (36.5%), hospitals are too expensive (35.4%), hospital staff not friendly (18.5%), not necessary for normal delivery (15.1%). | moderate |
| Ekirapa-Kiracho et al., 2011 [8] | Uganda | Quasi-experimental | Health-facility information system, surveys | Population of Kamuli District (680,500); Population of Pallisa District (480,000) | Access to vouchers for transport and delivery care (intervention vs. control areas) | | 1) Percent of women delivering at health facilities; 2) Percent of poor women delivering at health facilities | Descriptive statistics (preliminary analysis of larger study) | FBD increased from < 200 / month to 500+ / mo in the intervention areas following the introduction of vouchers; stayed < 200 / month in control areas. | moderate |
| Faye et al., 2011 [62] | Senegal | Cross-sectional | Household survey of women of reproductive age, 2006-2007 | 373 women who gave birth within past 12 months and delivered in a facility in past 5 years | Predisposing: age, education, marital status; Enabling: transport, incoming-generating activities, distance between home and facility; Previous delivery in facility; Quality/Satisfaction with care | | Place of delivery, facility (yes / no) | Multiple logistic regression analysis | 22% of the sample had home birth for most recent delivery. More frequent among those in polygamous unions, with no means of transport, who lived >5km from facility, who had a poor quality previous delivery and who had a previous delivery by a male attendant. | moderate |
| Fotso et al., 2008 [29] | Kenya | Cross-sectional | Nairobi Urban Health & Demographic Surveillance System (household interviews from 2 slum settlements of Nairobi), Facility Assessments, 2006 | 1927 women who had a pregnancy outcome in 2004-2005; 25 health facilities assessed | Maternal education, wealth, parity, location of residence, age, ethnicity | | 1) adequacy and quality of obstetric care provided in facilities; 2) ANC utilization; 3) Place of delivery (not in a facility vs. "appropriate" or "inappropriate" facilities) | Descriptive statistics, bivariate comparisons | 70% of deliveries from the settlements occurred in facilities; only 48% occurred in "appropriate" facilities (those with at least minimum standards). Differences by wealth, education, parity. Women reporting 4+ ANC visits more likely to delivery in "appropriate" facility. | moderate |
| Fotso et al., 2009 [28] | Kenya | Cross-sectional | Nairobi Urban Health & Demographic Surveillance System (household interviews from 2 slum settlements of Nairobi), Facility Assessments, 2006 | 1927 women who had a pregnancy outcome in 2004-2005; 25 health facilities assessed | Individual level: education, working status, ethnicity, household wealth, parity, pregnancy wantedness, ANC visits, whether advised to deliver at a facility at ANC; Community level: slum residence | | Place of delivery: didn't deliver in a facility, delivered in an inappropriate facility, delivered in an appropriate facility | Multivariate ordered logistic regression | 70% of deliveries from the settlements occurred in facilities; only 48% occurred in "appropriate" facilities (those with at least minimum standards). Multivariate: greater education, being currently employed, ethnic group, lower parity, being from a wealthy household, pregnancy wantedness, # of ANC visits, and being advised to deliver in a facility during ANC visits all linked to delivery in an appropriate facility. | strong |
| Fotso et al., 2009a [30] | Kenya | Cross-sectional | Nairobi Urban Health & Demographic Surveillance System (household interviews from 2 slum settlements of Nairobi), Facility Assessments, 2006 | 1927 women who had a pregnancy outcome in 2004-2005; 25 health facilities assessed | Women's autonomy (17-items: decision making, freedom of movement, overall autonomy); household wealth, women's education, parity, age at delivery, pregnancy wantedness, # of ANC visits, whether advised to deliver at a facility at ANC | | Place of delivery: didn't deliver in a facility, delivered in an inappropriate facility, delivered in an appropriate facility | Multivariate ordered logistic regression | Household wealth, education, wantedness of pregnancy, # of ANC visits, advice during ANC, and lower parity were linked to increased likelihood of FBD at an appropriate facility. Autonomy was not significant. Interaction effects suggested that the effect of autonomy varies by household wealth, with poor women having lower likelihood of FBD the higher their autonomy scores. | strong |
| Gabrysch et al., 2011 [69] | Zambia | Cross-sectional | Zambian Demographic Health Survey, 2007; Facility data from Zambian Health Facility Census, 2005; GIS data | 3682 rural births (between 2002 and 2007) with place of delivery data available; Facility data on EmOC capability on 665 facilities with at least 2 basic functions (minimum for inclusion) | Distance to the facility; Level of care at the facility | | Facility delivery | Multivariable multilevel logistic regression | 32.5% of births occurred in a facility, 0.4% were home deliveries attended by a nurse or midwife, 67.1% were neither in a facility nor attended professionally. Proximity to facility was strongly associated with facility birth, as was higher level of EmOC available within 15 km. In final model, 29% decrease in odds of FBD for each doubling of distance to the facility, 26% increase in odds of FBD for every step increase in level of EmOC. | strong |
| Gage, 2007 [64] | Mali | Cross-sectional | Mali Demographic Health Survey, 2001 | 6178 births to rural women between 1997 and 2001 | Area-level variables: services availability (health infrastructure, EmOC), physical accessibility (year-round roads, time to get to public transport, distance to facility), social environment (concentration of education, ethnicities, poverty, ANC uptake); Individual-level variables: household characteristics, mother's characteristics (education, duration of residence, barriers to medical care, exposure to counseling during ANC) | | 1) Receipt of ANC in first trimester; 2) 4+ ANC visits; 3) Attendance at delivery by trained medical personnel; 4) Delivery in facility | Multilevel logistic regression | 25.8% of births were assisted by a trained provider, 26.3% occurred in a facility. Financial barriers were cited most by women (58.1%) with distance (48.8%) and transportation (47.6%) also commonly cited. Multivariate analysis suggested distance barriers are important for both SBA and FBD, as well as living in close proximity to other women who had utilized ANC increased the odds of SBA and FBD. In high education areas, living there for 5+ years was linked with higher likelihood of delivery care. | moderate |
| Galaa and Daare, 2008 [34] | Ghana | Cross-sectional | Pregnancy case histories; qualitative interviews with staff, 2005-2006 | 496 pregnancy and delivery episodes in 3 districts in northern Ghana, unstated # of interviews | Region, rural/urban status, wealth, education, perceived quality of services, distance to facilities | | ANC utilization, Delivery in a facility, post-delivery services | Descriptive statistics | 63% of women said the most recent delivery was at a health facility: varied by district, rural/urban status, education. Half of home deliveries were done without a trained TBA or health professional. Women reported main reasons for FBD: confidence in facility as a place for safe delivery (23%), given referral or advice to deliver in a facility (26.5%), nearness to a facility (10%). Major reason for home delivery: taken by surprise in the middle of the night. | weak |
| Gyimah et al., 2006 [35] | Ghana | Cross-sectional | Ghana Demographic Health Survey, 2003 | 2084 women of reproductive age who have had births within past 3 years | Religion - denominational affiliation | | 1) Immunization against tetanus during most recent delivery; 2) Any ANC; 3) # of ANC visits; 4) Delivery in a facility | Poisson models, binary logit models | Overall, 41% of women delivered in a facility; 14% who identify w/ traditional religion, 32% Muslim, 42.9% Catholic, 49.6% Protestant, 52% other Christian religion delivered in a facility; Effect similar but attenuated when control for other covariates. (Bivariates showed Northern region, rural area, polygamous relationship, lowest income and lowest education most strongly linked to non-facility delivery,) | strong |
| Hodgkin, 1996 [59] | Kenya | Cross-sectional | Household survey, 1989 | 552 rural households, Nyanza province | Assets, measure of hunger in past 4 weeks, household size, insurance, head of household characteristics (note: no individual woman characteristics) | | Informal (home, TBA's home) vs. Formal (clinic, hospital) delivery | Binary Probit Regression | Half (52.3%) of deliveries occurred in informal setting. Deliveries in the formal sector were more likely among households with shorter distances to maternity beds, with health insurance, with non-male, non-farmer household head. Biggest predictor: distance to nearest maternity bed. | moderate |
| Hong et al., 2011 [52] | Rwanda | Cross-sectional | Rwanda Demographic Health Survey, 2005 | 5425 women aged 15-49 with live birth in past 5 years; 8715 births between 2001-2005 | Insurance status, birth order, woman's age at delivery, women's occupation, women's education, residence (urban/rural), province, household wealth index quintile | | 1) Deliveries at home, 2) Deliveries assisted by unskilled birth attendants or unassisted | Multilevel logistic regression | Overall, 61% of deliveries either unassisted or assisted by unskilled attendance; 38.6% with SBA. 44% births to insured mothers. 29.3% births delivered in health facility, 70.4% deliver at home. Multivariate analysis: births to insured women are significantly less likely to occur at home and significantly less likely to be assisted by unskilled birth attendant or unassisted. Odds decrease as wealth increases, odds higher in rural areas, varies by province. | moderate |
| Hounton et al., 2008 [36] | Burkina Faso | Cross-sectional | Facility assessments, modified DHS questionnaire in 2 districts | 81,536 births between 2002 and 2005 in 2 districts, 1 an intervention district for Skilled Care Initiative | # of health care providers per 10,000 population; physical inputs of health center, whether health center was involved in SCI intervention, distance from health center and main referral, maternal age at delivery, parity, multiple birth, education, asset quintile, year of birth | | 1) Institutional Birth Rates; 2) Cesarean Section rates | Logistic Regression | 38.4% of births took place in a facility; multivariate analysis: distance to health center, maternal age at delivery, education, assets most important to facility based delivery. (Note: # of providers per 10,000 was not significantly associated with FBD.) | strong |
| Houweling et al, 2007 [13] | Developing Countries | Cross-sectional | World Bank Country Reports, based on DHS in 45 countries, 1990-1998 | approximately 5,000 - 10,000 women per country | Wealth index based on multiple indicators, divided into quintiles | | 1) Professional delivery attendance, 2) Professional ANC, 3) Childhood immunization, 4) Treatment for diarrhea, 5) Treatment for Acute Respiratory Infections | Calculated Rate Ratios: ratio of use among the richest quintile vs. the poorest quintile | Among the richest quintile, use of professional delivery reached 80% or higher, although among the poorest it is below 30% in many countries. Absolute poor-rich gap is largest in the public sector, relative poor-rich inequalities are larger in private sector. SBA highest among urban rich, lowest among rural poor. "Professional delivery care is nearly synonymous with facility-based care in most countries with a few exceptions." | moderate |
| Idris et al., 2006 [18] | Nigeria | Cross-sectional | Interview-administered survey in semi-urban settlement in northern Nigeria, 2003 | 496 women between 14 and 50 years old who had delivered at least once | Maternal age, education, age at first pregnancy, ANC attendance, father's occupation and education | | 1) Delivery location, 2) Delivery supervision | Descriptive statistics, bivariate comparisons | 70% had home deliveries, 78% had unsupervised deliveries (deliveries not supervised by an SBA). Main determinants of place of delivery were mother's education level, husband's occupation, age at first pregnancy. | weak |
| Kruk et al., 2007 [23] | 42 low-income countries | Cross-sectional | DHS data, WHO national accounts database (1998-2004), World Bank Development report (1993) | 42 low-income countries with indicators of Maternal Health utilization in DHS | Gov't health expenditure as a percentage of total health expenditure; per capital health expenditure; female literacy | | 1) National Rates of ANC usage; 2) National Rates of SBA; 3) National Cesarean Section Rates | Multivariable regression | In 42 low-income countries with adequate data, median rate of SBA was 49.8%. Gov't share of health care spending was significantly associated with SBA utilization rates. Female literacy rates and total expenditure per capita were also significantly associated with SBA. | strong |
| Kruk et al., 2008 [37] | 45 developing countries | Cross-sectional | DHS data, 1990-2001 | 45 developing countries w/DHS data on SBA and wealth indicators | Health care expenditures per capita, proportion of population in poverty, ratio of 5th grade completion between richest and poorest wealth quintile, rate of 5th grade completion by women of reproductive age (15-49) | | Ratio of SBA utilization by the poorest vs. the richest population quintile (equitable would be 1, w/lower utilization among poor <1) | Multivariable regression | Women in highest income group had higher rates of SBA than women in the poorest group. In bivariate comparisons, there was significant link to SBA and health expenditures per capita, ratio of 5th grade completion, rate of 5th grade completion. In multivariable regression, only significant factor was proportion of women in a country completing the 5th grade. 3-way interaction term was also significant in other models. | strong |
| Kruk et al., 2009 [72] | Tanzania | Population-based discrete choice experiment (DCE) | Interviews with women from randomly selected households, 2007 | 1203 women who completed 9611 DCE cards | Attributes of facilities: distance, transport, provider type, attitude of provider, drugs and equipment availability, cost | | Attributes of desirable facilities | Hierarchical Bayes Modeling | 60.8% of women in the sample who'd had a pregnancy in the previous 5 years delivered at home; 36.5% delivered in a facility. The most important facility attributes were a respectful provider attitude and availability of drugs and medical equipment. Policy modeling suggested if these attributes were improved in existing facilities, FBD rates could rise 43% to 88%. | strong |
| Kruk et al., 2010 [60] | Tanzania | Cross-sectional | Population-based household survey, 2007 | 1205 women from rural Tanzania | Individual level: age, poverty, education, health insurance, women's knowledge of benefit of SBA, parity, age at delivery, ANC visits, perception of quality of doctors and nurses / care at nearest facility; Village level: presence of clinic, aggregated individual responses to get village level responses about attitudes toward FBD, ANC, skill of doctors, skill of TBAs, quality of facility | | Facility delivery | Multilevel logistic regression | Both individual-level and village-level variables were significant predictors of FBD. Individual level: community health insurance, no previous births, 'facility delivery is important', 'excellent quality' at local facility. Village level: % of village who ....stated FBD was important, attended 4+ ANC visits, stated 'excellent quality' at nearest facility, agreed doctors and nurses have good skills, agreed TBAs have good skills. (Latter: the higher, the lower FBD.) | strong |
| Kyomuhendo, 2003 [67] | Uganda | Cross-sectional, mixed methods | Focus groups, key informant interviews, survey among women with >1 previous birthing experience, maternal death inquiries, 2000-2001 | 808 women with previous deliveries, 24 FGDs with 240 participants (both men and women), 20 IDIs with purposively selected elders, TBAs, HCPs | Age, marital status, education, occupation; attitudes, beliefs about childbirth and pregnancy complications | | Why women avoid FBD, even in face of serious complications | Descriptive statistics, qualitative analysis | Pregnancy is seen as a journey on a dangerous path, to seek external help is to stumble. To die is to be a "hapless victim of a thorn-strewn path". Even if symptoms become severe, "a proper woman" would not communicate that pain to just anyone. 58% of women delivered their first child outside a health facility. Although 65% of women said they experienced pregnancy-related problems, only 10% consulted HCP. 42% consulted no one. FBD described as impersonal, insensitive, and "you are treated like a child or a fool." | weak |
| Letamo and Rakgoasi, 2003 [19] | Botswana | Cross-sectional | Botswana Family Health Survey, 1996 | 1184 women from 15-49 years old who had at least 1 pregnancy in previous 5 years | Individual factors: age, parity, education, marital status, place of residence; Household factors: SES | | Institutional Delivery, ANC, qualified delivery assistant, tetanus vaccine during pregnancy, postnatal visits | Bivariates and multivariable logistic regression | In multivariate model, younger women less likely to deliver outside a facility, women with one child were more likely to delivery outside a facility> Women with no formal education, low SES, rural residence more likely to delivery outside a facility. Unqualified birth assistance most likely among young women, women with 2-3 children, village residence, and low SES. | strong |
| Magadi et al., 2000 [25] | Kenya | Cross-sectional | Kenya Demographic and Health Survey, 1993 | 5,290 births with complete information from mothers, households, and service availability questionnaire | Individual, household, community level factors: Individual (education, SES, rural/urban, region, ethnicity, birth order, desirability of pregnancy, family planning practice, ANC visits), household (time, distance to health facility, SES), community (time, distance to health facility) | | Who attended delivery: medical personnel, TBA, relative/unskilled, no one | Multi-level Logistic and Multi-level Multinomial Regression | Overall, 58% of deliveries occurred outside facilities, 42% in facilities. 12% of deliveries were attended by a doctor, 31% nurse/midwife, 20% TBA, 27% relative/unskilled, 11% no one. FBD more likely among those with higher education, higher SES, living in urban areas, living in the Central province, of Kalenjin ethnicity, with a desired pregnancy and births lower in the birth order. FBD also more likely with more ANC visits, shorter distances to facility. | moderate |
| Magadi et al., 2007 [20] | Sub-Saharan Africa | Cross-sectional | DHS data from 21 countries, late 1990s-early 2000s | DHS data related to maternal health services from 21 countries in Sub-Saharan Africa | Controlled for parity, premarital births, education level, urban/rural status, while focusing if diff rates among teenage mothers (15-19), mothers 20-34 and mothers 35+. Also at country level: Gross National Income per capital, per capital health expenditures, health expenditures as a % of GNI, female literacy | | ANC utilization (% with late start, % with inadequate visits); Delivery care (non-facility delivery, unskilled attendance) | Bivariates and multivariable logistic regression | Across 21 countries in SSA, teenage mothers are more likely to have non-facility delivery and unskilled attendance than women 20-34 or 35+, even after controlling for parity, premarital birth, education, and urban/rural residence. Significant difference in 13/21 countries for FBD, significant difference in 7 of 21 for skilled attendance. In pooled analysis, age, rural residence, education, birth order, GNI per capita associated with non-FBD. Similar for skilled attendance except health expenditures per capita instead of GNI. | moderate |
| Martey et al., 1995 [80] | Ghana | Cross-sectional | Population-based survey, 1990 | 1200 women between 15 and 49 from Ejisu District outside Kumasi | Age, education level, occupation, marital status, age at first marriage | | ANC, place of delivery | Descriptive statistics | 22% of women delivered last infant in a hospital, 33% delivered in a health center, 28% delivered in a government maternity home, 13% with a TBA, 3.7% with "other". In total, 83.3% delivered in a facility, "at odds with Ministry of Health Annual Reports." | weak |
| Mbonye and Asime, 2010 [53] | Uganda | Cross-sectional | Health facility survey of 553 facilities, year unstated | 553 facilities from 54 districts in Uganda | # of staff at facility, availability of equipment, delivery room, accommodation for staff, availability of electricity / water, ambulance for referral, radio communication | | Facility delivery (by level of facility) | Descriptive, Bivariates, Poisson Regression | 194,029 deliveries recorded - 60.7% occurred in hospital, 17% occurred in health center IVs, 22.3% occurred in health center IIIs. Significant variables in the multivariate analysis: region, electricity, running water, radio communication, and accommodation for staff were the factors most likely to attract women to deliver at facilities. Availability of comprehensive EmOC and basic EmOC had highest chances of attracting women to deliver at health facilities. | weak |
| Mekonnen and Mekonnen, 2003 [39] | Ethiopia | Cross-sectional | Ethiopian Demographic Health Survey, 2000 | 7,987 women who had at least 1 child < 5 yrs. old at time survey was fielded | Maternal age at birth, parity, number of children < age 5, education, marital status, work status, religion, residence, year of birth of child | | Antenatal care, delivery care | Multivariate logistic regression | Only 6.2% of women delivered in 5 yrs. prior to survey were assisted by health professionals. Substantial variation by residence, parity, education, religion, and marital status; residence, education, parity and # of children < age 5 served as independent predictors of delivery services. | moderate |
| Mills et al., 2008 [65] | Ghana | Cross-sectional | Interviews from women identified through Navrongo DSS, 2005-2006 | 3,433 women who had delivered in 2004; info on 259 community facilities | Assessed a total of 24 individual-level predictor variables (e.g. age, education, marital status, etc.) and 7 community-level predictor variables (e.g. urban/rural, availability of public transport, etc.) | | Use of health professionals to assist in birth | Multilevel logistic regression | 98% of women received ANC; only 38% delivered with assistance of health professional. In multivariate analysis, physical access factors and community perception of access to nearest facility were strongly associated with FBD. Women who knew it was free were 4.6 times more likely to use health professionals. Lower parity, greater education linked to higher likelihood of FBD. Community perception of quality of care was not related. Article shows strength of community-level factors relative to individual-level factors. | strong |
| Montagu et al., 2011 [57] | Multiple Countries | Cross-sectional | DHS data from 48 countries, 2003-2011 | DHS data from 48 countries, focus on 27 countries in Sub-Saharan Africa, focus on maternal deliveries | Wealth quintile, cost barriers, access barriers, other barriers | | Delivery location (public facility, private facility, home), who attended delivery | "Secondary analysis" | Richest women most likely to deliver in a gov't facility. In Sub-Saharan Africa the poorest women were 3x more likely to report giving birth at home than the richest women. 77.7% vs. 22.4% . Both groups had similar rates of non-attendance by a professional. 56% of home births to poor women were unattended - 40% of home births to rich women were unattended. Access cited more often than cost as a reason for non-FBD, among rich and poor women most often cited reason: "It's not necessary." | moderate |
| Mpembeni et al., 2007 [21] | Tanzania | Cross-sectional | Interviews with women in Mtwara rural District Hospital, year not stated | 974 women who gave birth within 1 year prior to study | SES, knowledge of pregnancy danger signs, age, years in school, marital status, household size, parity, distance to facility | | Use of skilled care at delivery | Multiple logistic regression analysis | 99.8% attended ANC at least once - 46.7% delivered in a facility, 44.5% were assisted by a skilled attendant. Distance, discussion with male partner on place of delivery, advice to deliver in a facility during ANC, and knowledge of pregnancy risk factors were significantly linked to use of skilled care at delivery. Also, age, education, and marital status. | strong |
| Mulogo et al., 2006 [24] | Uganda | Cross-sectional | Community-based survey in 2 sites, 2002 | 415 respondents from 2 rural districts in Uganda, 1 site subject to more intense intervention | Completion of a birth plan, constructs of fear, perceived self-efficacy, perceived response efficacy | | Delivery in a facility | Bivariates and multivariable logistic regression | Significant relationship found between completion of a birth plan and delivery in a health facility. Education and marital status was associated with the completion of birth plans, but marital status was not linked to FBD. Fear of home delivery linked to completion of birth plan, Fear of use of facility also linked to completion of a birth plan. | weak |
| Mwaniki et al., 2002 [22] | Kenya | Cross-sectional | Interviews via structured QQ, focus group discussions, 2000 | 200 mothers bringing children to welfare clinic at 1 of 4 rural health centers in Mbeere District | Marital status, religion, age, number of children, education, occupation, distance to health facility, time taken, mode of transport | | ANC, utilization of maternity services | Bivariate analysis | Age of mother, distance to facility, number of children all influenced use of maternity services. (More children = lower utilization; >5km = lower utilization; older mothers less likely to deliver in a facility.) | weak |
| Nilses et al., 2002 [81] | Zimbabwe | Cross-sectional | Interviews among women in randomly selected villages in Southern Zimbabwe, 1992-1993 | 889 women aged 15-44 years old; 3601 pregnancies | N/A | | Reproductive outcome, booking and time of ANC, morbidity and utilization of ANC, care and complications of delivery | Descriptive statistics | 15% of women delivered at home; 1/3 of which were assisted by a TBA. 27% delivered in a clinic; 58% delivered in a hospital (total of 85% delivering in a facility). | weak |
| Nuwaha & Amooti-Kaguna, 1999 [40] | Uganda | Cross-sectional | Interview data, 1997 | 211 women in rural Uganda who had a delivery in the past year | Maternal SES, reproductive variables, self-efficacy variables | | Home Delivery | Stepwise multivariate analysis | Univariate: variables that favored home delivery were region, no primary education for the mother, ethnicity, religion, no secondary education for the father, maternal parents being peasants, living > 5 km from maternity center, etc. (Note: 14 variables tested among 211 women) Multivariate: Not being from Kyotera County, father being a peasant, previous delivery at home, not being from a high social class. Highest risk factor for home delivery was a previous home delivery. | moderate |
| Ochako et al., 2011 [31] | Kenya | Cross-sectional | Kenya Demographic and Health Survey, 2003 | 1675 women aged 15-24 who delivered within past 3 years | Education, household wealth, urban-rural residence, ethnicity, parity, age at birth of last child, marital status | | Timing of first ANC visit, type of delivery assistance (none, TBA, SBA) | Multivariate ordered logistic regression | Major finding: association between early ANC and use of SBA. Place of residence, wealth, education, ethnicity, parity, marital status and age at birth of last child had strong influence on both first ANC visit and the type of delivery assistance received. | strong |
| Oguntunde et al., 2010 [41] | Nigeria | Cross-sectional | Household survey in one of 3 purposively selected communities, 2008 | 332 women aged 15-49 who delivered within the past 24 months | Age, marital status, occupation, religion, educational status, parity | | ANC attendance (yes / no, number of visits); SBA | Descriptive statistics, bivariate comparisons | 86.7% of deliveries occurred at home; 11.7% in a health facility; 1.5% in another location. 14% of births had SBA, 35.8% TBA, 46.1% friends / family / self, 3.9% others. FBD and SBA increased with increasing education. | weak |
| Olusanya et al., 2010 [42] | Nigeria | Cross-sectional | Interviews with mothers attending community health centers for immunizations, 2005-2007 | 6465 women with 6558 infants | Sociodemographic and obstetric variables, including: maternal age, ethnicity, marital status, parity, religion, education, occupation, type of accommodation, quality of accommodations, social class of mothers, history of ANC, use of herbal drugs, prior C-section, multiple gestation | | Place of delivery | Bivariates and multivariable logistic regression | 51.4% delivered outside hospital (81.1% of that group had no SBA). Non-hospital delivery or presence of unskilled attendance delivery was associated with teen mothers, Muslim religion, low or middle social class, use of herbal drugs in pregnancy. Non-hospital delivery also associated with ethnicity, l/o tertiary education, l/o fulltime employment, accommodation with shared sanitation facilities, and multiparity. Key point: availability of and proximity to facilities isn't a guarantee of uptake of maternity services. | strong |
| Onah et al., 2006 [48] | Nigeria | Cross-sectional | Interview survey among women who had delivery w/in past 3 months, 2004 | 1098 women in Southeastern Nigeria who had delivered within past 3 months | Women's perceived factors determining choice (21 factors); maternal factors (e.g. age, education, marital status, etc.); paternal factors (e.g. education, occupation, etc.); SES; Household location; Religion | | Place of delivery (7 different options articulated: home, TBA's home, spiritual houses, maternity homes / primary care centers, general hospitals, teaching hospitals, private hospitals | Bivariate analysis | 52.9% delivered outside institutions; 47.1% delivered in a health institution. Major influencers of both groups: cost, competence of delivery attendants, promptness of care. Major influences for FBD: competence of the doctor, nearness of the place of delivery, friendliness of staff, availability of equipment, presence of OB/GYN, 24-hour presence of doctors, team work among doctors, availability of facility for C-section, availability of health education. Urban dwellers and Christians more likely to deliver in a facility. | strong |
| Osubor et al., 2006 [71] | Nigeria | Cross-sectional, mixed methods | Survey among randomly selected women in rural Nigeria; FGDs with community women and HCWs, 1999 | 225 randomly selected mothers (15-49 yrs. old); 6 FGDs: 4 for women, 2 for HCPs | Age, occupation, religion, education, marital status, number of children | | ANC, FBD | Descriptive statistics, bivariate comparisons, qualitative analysis | Preferred place of delivery among 225 women: gov't clinic (15.7%), private maternity home ((37.3%), with TBA (25.5%), church (7.8%), other (13.7%). Most common reason for NOT delivering in clinic: uncertainty about availability of trained staff (31.4%), perception of poor quality of services (24.3%), high cost (19.2%), other (11.8%). Among 81 who delivered in past year, 49.4% delivered in a private maternity home, 42% delivered with a TBA. Qualitative data suggest that perceived etiology of pregnancy problems influenced care seeking: "Traditional/ Spiritual problems" treated differently than "normal / physical" problems. | weak |
| Penfold et al., 2007 [9] | Ghana | Cross-sectional, assessing pre-post intervention | Interviews with women who had delivered before / after fee exemption policy, 2006 | 2,922 women ages 15-58 yrs. who had delivered either before or after fee exemption policy; Central and Volta regions | Poverty quintiles, education, whether delivery occurred before or after fee exemption, enumeration area | | Place of delivery, who attended delivery | Multilevel logistic regression | More deliveries occurred at home in Volta region (49.3%) than in Central region (32.9%). Most commonly reported facility for delivery was hospital (30.2 in Central, 32.5% in Volta). Midwives were most common professional attendants (56% in Central, 49% in Volta). Strong association between SBA and FBD, no need for separate analyses. FBD increased after fee exemption policy (12% in Central, 5% in Volta). Increase in Central region mostly attributable to increase in health center deliveries. Increases linked to education and wealth (biggest increases seen among women with least education, from lowest wealth quintile). | moderate |
| Rockers et al., 2009 [15] | Tanzania | Cross-sectional | Population-based household survey, 2007 | 1204 women in rural Tanzania who'd given birth within the past 5 years | Frequency of ANC, characteristics of ANC, household characteristics, childbirth history, knowledge and perceptions of local healthcare system | | Facility delivery | Bivariates and multivariable logistic regression | Home delivery: 64% vs. FBD: 36%. In bivariate analysis, FBD more likely among women < 25 yrs. old, who had < 2 children, lived <5km from facility, or stated a belief that facility is important. Similar in multivariate analysis. Also in bivariates, FBD more likely among women with 4 or more ANC visits or who visited a gov't health center or mission facility (as opposed to gov't dispensary). | strong |
| Smith and Sulzbach, 2008 [56] | Senegal, Mali, Ghana | Cross-sectional | Household surveys conducted by USAID-funded Partners for Health Reform Plus, 2004 | 775 women in Mali, 293 women in Ghana, 191 women in Senegal; all split between community-based health insurance and not; delivered in past year or were pregnant | Membership in voluntary community-based health insurance scheme, SES, age, parity, previous birth experiences, parity | | Initiation of ANC, 4+ ANC visits, delivery at modern health facility | Descriptive statistics and multiple regression | Membership in voluntary CBHI scheme was significantly associated with increased FBD: Senegal (71.1% among nonmembers, 93.1% among members with delivery services covered); Mali (64.6% among nonmembers, 93.7% among members); Ghana (64.5% among nonmembers, 74.8% among members). In multivariable analysis, SES was significantly associated with FBD in Ghana, urban status was significant in all three countries, CBHI membership increases FBD in Mali and Senegal. | moderate |
| Spangler and Bloom, 2010 [50] | Tanzania | Mixed Methods | Household survey / interviews, Ifakara DSS data, 2007 - 2008 | 1155 women from the DSS / quantitative survey; 48 qualitative interviews with women who delivered in the past year | Ethnicity, religion, marital status, education, household head occupation, household assets, distance to facility, referred at ANC, facility in village, district of residence, perceived problems, parity | | Use of 'biomedical obstetric care' (facility-based delivery) | Logistic regression, qualitative analysis | 64% of women used 'biomedical obstetric care' (FBD). Of the 668 who reported FBD, 624 reported being attended by a biomedical provider. In regression, ethnicity, household assets, distance to facility, district of residence, facility in village, referred at ANC, perceived problems during pregnancy, parity, and month of first ANC visit all linked to FBD. In qualitative analysis, motivators differed by income / asset level, including desire to appear modern, fear of being shamed, affordability of all associated supplies, other factors playing important role. | moderate |
| Stanton et al., 2007 [10] | Multiple Countries | Longitudinal look at trends in SBA | Nationally representative population-based surveys, 1986-1994 for 1990 estimate, 1996-2003 for 2000 estimate | 73 countries' data on percent of deliveries with SBA | Age, parity, country | | % births with SBA, Difference between 1990 and 2000 | Data compilation, trend analysis | Skilled birth attendance increased in the developing world from 45% to 54% between 1990 and 2000. (Note: in Sub-Saharan Africa it went from 39.4% to 39.8%.) Increases are largely due to increases in the use of doctors (based on a restricted sample of countries). But in Sub-Saharan Africa, percent increase in using doctors was actually negative (-2.8%). Increases were seen among the use of nurses / midwives/ auxiliaries (5.6%). Oldest women are least likely to have SBA in Africa, and higher parity = lower SBA rates. | weak |
| Stephenson et al., 2006 [14] | Kenya, Malawi, Tanzania, Burkina Faso, Ivory Coast, Ghana | Cross-sectional | DHS data from 6 Africa countries 1998-2000; Contextual data from Geographic Information Systems | Kenya (3058 women); Malawi (6318 women); Tanzania (1710 women); Burkina Faso (3167 women); Ivory Coast (1131 women); Ghana (1785 women) | Individual / household level: age, parity, marital status, education, religion, exposure to family planning information, ANC during last pregnancy, previous delivery in a facility, household assets, urban residence; Community level: mean # of children per primary sampling unit (PSU), PSU-level approval of family planning, PSU-level of female education, PSU-level of maternal health service use, PSU rainfall | | Childbirth in a health facility | Multilevel modeling, including multilevel logistic regression | Few variables proved to be significantly associated with FBD across all six countries. (e.g. age linked in Malawi, Tanzania, and Kenya, not in Burkina Faso, Ivory Coast, or Ghana; education (secondary or higher) linked in Malawi, Kenya, Burkina Faso and Ghana, but in Tanzania only women with primary education were more likely than women with no education to have FBD. No relationship in Ivory Coast.) In Ghana, urban status, being older or younger than 20-29, lower parity, being non-Muslim, having had 4+ ANC visits all linked to greater FBD. Main point: Marked variability across 6 countries in Western and Eastern Africa. | moderate |
| Tann et al., 2007 [70] | Uganda | Cross-sectional | Community-based survey, 2004 | 413 women from a semi-urban community in central Uganda who had a pregnancy within the past 5 years | (Predictor variables not utilized for place of delivery) Year of pregnancy, age, gravidity, education, possessions, electricity, crowding at home. | | ANC attendance, delivery location, delivery practices | Descriptives on place of delivery; logistic regression on ANC attendance and delivery practices | 11% of women delivered at home without a trained assistant, 5% used TBA, 20% used public clinic, 63% used hospital. Those delivering at home cited financial and transportation barriers. | weak |
| Telfer et al., 2002 [68] | Gambia | Cross-sectional | Surveys conducted among women identified through Farafenni demographic surveillance site, 1998-1999 | 623 women who had recently given birth in rural Gambia | Parity, illustrated by primipara vs. 6+ deliveries | | ANC visits, content of ANC visits, place of delivery | Descriptive statistics | 10.6% of women delivered at a major health center or hospital, 7.2% delivered at a dispensary, 78.6% delivered at home or at their mother's home. 4.9% delivered by themselves, 24.5% were assisted by a relative, 51% were assisted by a TBA, 19.3% were assisted by a nurse. Only 12 of 111 women who delivered at a facility said they made the decision on their own. Rest said they needed to seek permission from husband, TBA, mother, or mother-in-law. | weak |
| Uyirwoth et al., 1996 [66] | Lebowa, Swaziland | Cross-sectional | Household survey, 1992 | 210 women who had delivered within the past 12 months | Parity, ANC attendance, reasons for home delivery, perceived dangers of home delivery, tetanus immunization, post-natal care | | ANC, place of delivery | Descriptive statistics | 74.6% of women delivered in a facility. 26.3% delivered at home - cited lack of access to facility, lack of money, negative staff attitudes, precipitate labor, cultural beliefs and fear of episiotomy as barriers to FBD. | weak |
| Uzochukwu et al., 2004 [43] | Nigeria | Cross-sectional | Household survey, 2002 | 393 women aged 15-49 from southern Nigeria | Age, marital status, occupation, religion, residence, parity, social class, choice of health care resource, reasons for use and non-use of services, willingness to use maternity waiting homes | | Utilization of ANC, delivery services, post-natal services | Descriptive statistics, bivariate analysis | With regard to delivery services, bivariate comparisons indicate that younger women, those with no education, those living in a rural area, and those in lower social classes were less likely to use delivery services. | weak |
| van den Broek et al, 2003 [44] | Malawi | Cross-sectional | Household survey, year not stated | 59,248 men and women in 21,249 households; 20,416 women over age 10 provided obstetric history | Women's education, distance from health center, household type | | Pregnancy outcome, maternal morbidity, estimates of maternal mortality and perinatal mortality, type of assistance received at delivery | Bivariates and multivariable logistic regression | 63% of births had SBA (7% physician, 56% nurse / midwife). TBAs supervised 18.6% of deliveries. Unskilled relative (usually grandmother) supervised 15.9% of deliveries. No assistance in 2.5%. As education increases, SBA increases. As distance increases, SBA decreases. | weak |
| van den Heuvel et al., 1999 [58] | Zimbabwe | Cross-sectional | Community-based survey, 1996 | 235 women who had delivered in past 3 years, rural Zimbabwe | Maternal age, marital status, religion, education, work, SES, ANC card, obstetric history, use of family planning, pregnancy complications, # of antenatal visits, use of maternity waiting shelters | | Non-use of antenatal care facilities, hospital delivery | Logistic Regression | 66% of women delivered in a hospital, 11% in a rural clinic, and 22% at home or on the way to a facility. Use of maternity waiting shelters and complications during last pregnancy were strongest predictors of hospital delivery; unemployment and being without a husband were associated with non-facility delivery. | moderate |
| Wanjira et al., 2011 [45] | Kenya | Cross-sectional | Hospital-based survey of women who recently delivered, 2009 | 409 women visiting well child clinic reported 1170 deliveries in preceding 2 years | Age, total number of deliveries, maternal education, perception of home vs. hospital attendants, satisfaction, knowledge | | Delivery by skilled birth attendant | Binary logistic regression | 48% of women delivered in a facility, 48.2% had skilled birth attendant. Significant association between first and last place a woman delivered - a woman was 3.9 times more likely to deliver in the same place as her previous birth than to deliver somewhere different. In multivariate analysis, women with unskilled assistance were more likely to have < 3 yrs. education, 3+ lifetime deliveries, perceived similarity of skilled and unskilled birth attendance, and low knowledge of safe delivery. (Note: "In Kenya, utilization of maternal health services differs from district to district and community to community.") | strong |
| Wilkinson et al., 1997 [82] | South Africa | Cross-sectional | Survey of mothers in KwaZulu-Natal | 480 mothers of children aged 12-35 months | N/A | | Proportion of women receiving ANC and delivering in a facility | Descriptive statistics | 83% of women delivered in a health facility; 91% had attended ANC. | weak |
| Woldemicael, 2010 [46] | Eritrea and Ethiopia | Cross-sectional | Eritrea Demographic Health Survey, 2002; Ethiopia DHS, 2005 | 5,730 currently married women in Eritrea, 9,060 currently married women in Ethiopia, all who had last birth within past 5 years | Women's autonomy (participation in decision making, decisions of visiting families or relatives, attitudes toward partner violence), socioeconomic factors (education, employment, type of residence, age) | | Maternal health care utilization (at least 4 ANC visits, delivery care (facility or home, regardless of attendant)) | Multivariate logistic regression | Autonomy varies across Ethiopia and Eritrea. In multivariate analysis across both countries, delivery in a facility was more likely among women who made decisions on large household purchases jointly with husband, had higher levels of education, whose husbands had higher levels of education, were working / employed and who were not living in a rural area. | strong |
| Zere et al., 2011 [47] | Namibia | Cross-sectional | Namibia Demographic and Health Survey, 2006-2007 | 10,000 households across Namibia (exact number of births not reported) | Region, place of residence (urban/rural), wealth, education, head of household, insurance coverage, socioeconomic variables | | Delivery by skilled birth attendant | Linear probability model, decomposition analysis, developing a concentration index to measure wealth-related inequality | 80.3% of deliveries were attended by skilled health providers, SBA in richest quintile is about 70% more than that of the poorest quintile; higher among educated women and urban women. Biggest drivers in inequality of SBA: income, education, and urban residence. | moderate |
